# THE INTERACTIVE EFFECTS OF EDUCATION AND SOCIAL SUPPORT ON COGNITION IN AFRICAN AMERICANS

**DOI:** 10.1093/geroni/igac059.2076

**Published:** 2022-12-20

**Authors:** DeAnnah Byrd, Yanping Jiang, Samuele Zilioli, Peter Lichtenberg, Roland J Thorpe, Jr., Keith Whitfield

**Affiliations:** Arizona State University, Phoenix, Arizona, United States; Rutgers, The State University of New Jersey, New Brunswick, New Jersey, United States; Wayne State University, Detroit, Michigan, United States; Wayne State University, Detroit, Michigan, United States; Johns Hopkins University, Baltimore, Maryland, United States; University of Nevada, Las Vegas, Las Vegas, Nevada, United States

## Abstract

This study examines whether the effects of receiving or providing social support on cognition differ by education. Data from 602 African American adults (48-95 years) enrolled in the Baltimore Study of Black Aging—Patterns of Cognitive Aging were analyzed using multiple linear regression. We found no main effects of receiving or providing social support on global cognition. However, a significant moderation effect was observed for memory, such that received social support was more strongly associated with higher working memory among less-educated individuals than those with high levels of education, adjusting for age, sex, marital status, chronic health conditions, and depressive symptoms. Study findings demonstrate that social support and education have joint effects on memory outcomes, highlighting the importance of considering psychosocial protective factors that might alleviate, reduce, or even eliminate cognitive health disparities in African Americans.

